# Supplementary material for: Pathogenesis of hypertension in a mouse model for human CLCN2 related hyperaldosteronism
Source: Nat Commun. 2019 Oct 15;10:4678. doi: 10.1038/s41467-019-12113-9 (PMC6794291; doi:10.1038/s41467-019-12113-9)
Supplement: Supplementary file 1 — Supplementary Information [file 41467_2019_12113_MOESM1_ESM.pdf]

Pathogenesis of hypertension in a mouse model for human *CLCN2* related hyperaldosteronism

Göppner et al.

Supplementary Figures 1-10

Supplementary Table 1

Supplementary references

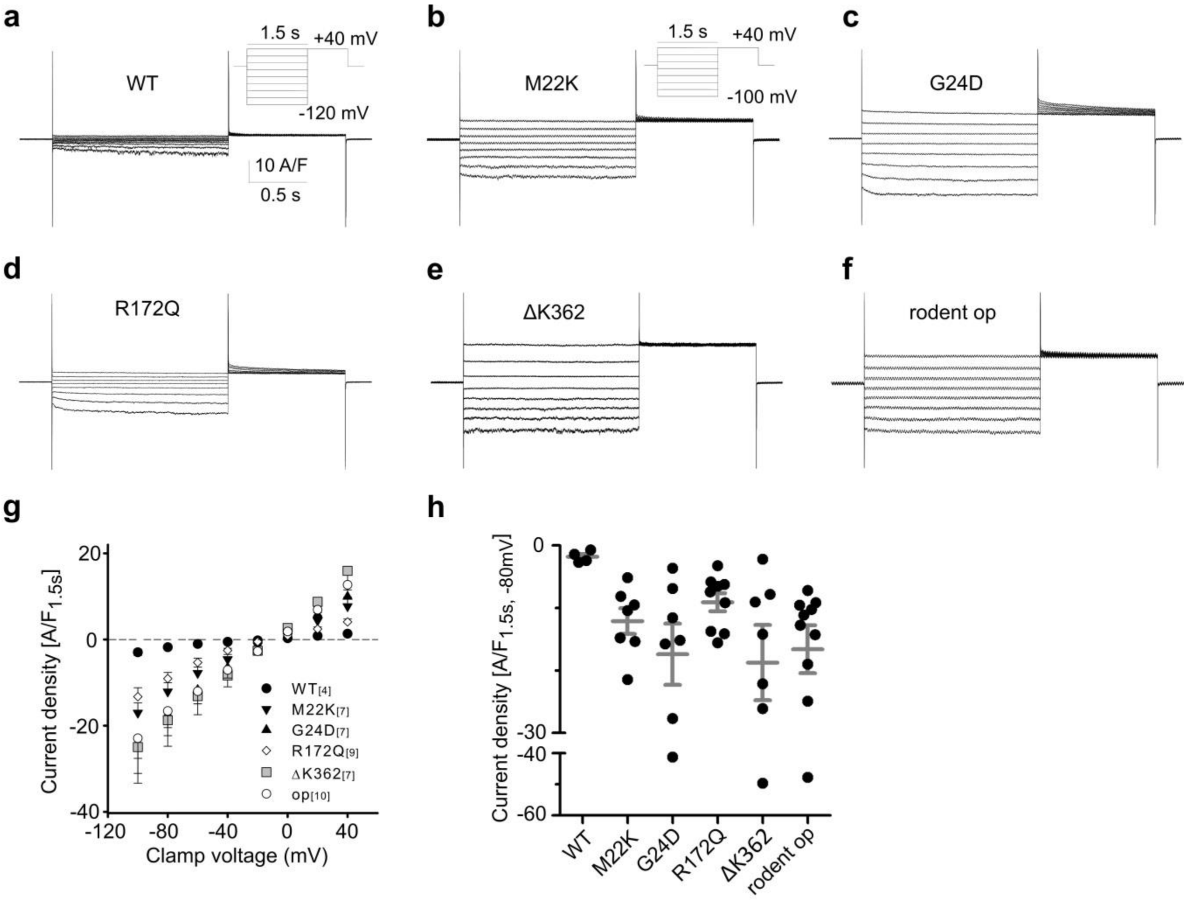

Supplementary Figure 1. Electrophysiological characterization of human PA-associated *Clcn2* gene variants and the op allele in transfected HEK cells. **a-f** Representative chloride current density traces from amphotericin-perforated patch-clamped transfected HEK cells. (1.5 s 20 mV steps applied from +40 mV to either -120 mV (WT) or -100 mV (mutants), with a final 1 s clamp at +40 mV). **g, h** Plot of mean current densities measured at the end of the voltage pulse (1.5 s) as function of voltage (**g**) and individual current densities (**h**) measured at -80 mV of experiments performed in (**a-f**). The number of cells measured indicated in parenthesis are from 4-10 transfections. Data are presented as means ± SEM.

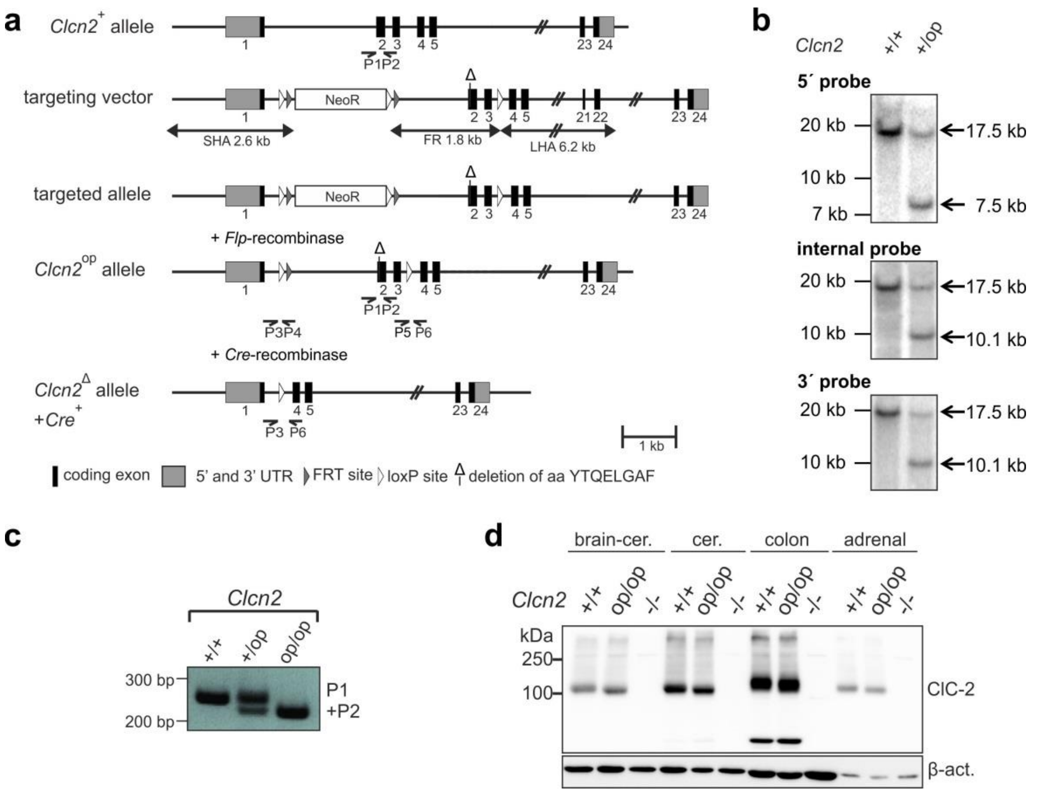

Supplementary Figure 2. Generation and characterization of the ClC-2 gain of function mouse model. **a** To generate the ClC-2 gain-of-function ('open') mouse model (*Clcn2*$^{op/op}$), a deletion of amino acids YTQELGAF (and an additional change of two amino acids to create an EcoRV restriction site for Southern blot analysis) were introduced in the targeting vector. Additionally, a neomycin resistance cassette (NeoR) for ES cell selection flanked by FRT sites and loxP sites flanking exons 2 and 3 were inserted. The NeoR cassette was removed by FLP-*FRT* recombination. **b** Southern blot analysis comparing the first generation of *Clcn2*$^{+/op}$ to *Clcn2*$^{+/+}$ mice ascertained the correct insertion of the targeted allele. **c** PCR genotyping of *Clcn2*^{op} mice using primers (P1 and P2) indicated in panel (**a**). **d** Representative Western blot analysis of ClC-2 of membrane fractions isolated from brain without cerebellum (brain-cer.), cerebellum (cer.), distal colon and adrenal glands of male *Clcn2*$^{+/+}$, *Clcn2*$^{op/op}$ and *Clcn2*$^{-/-}$ mice. Brain and colon were chosen because they display robust expression of ClC-2 and have been studied previously (and in this work) by techniques that included ClC-2 immunohistochemistry[1-3]. Equal amounts of protein were loaded with β-actin serving as loading control. We repeated this analysis with two other mice.

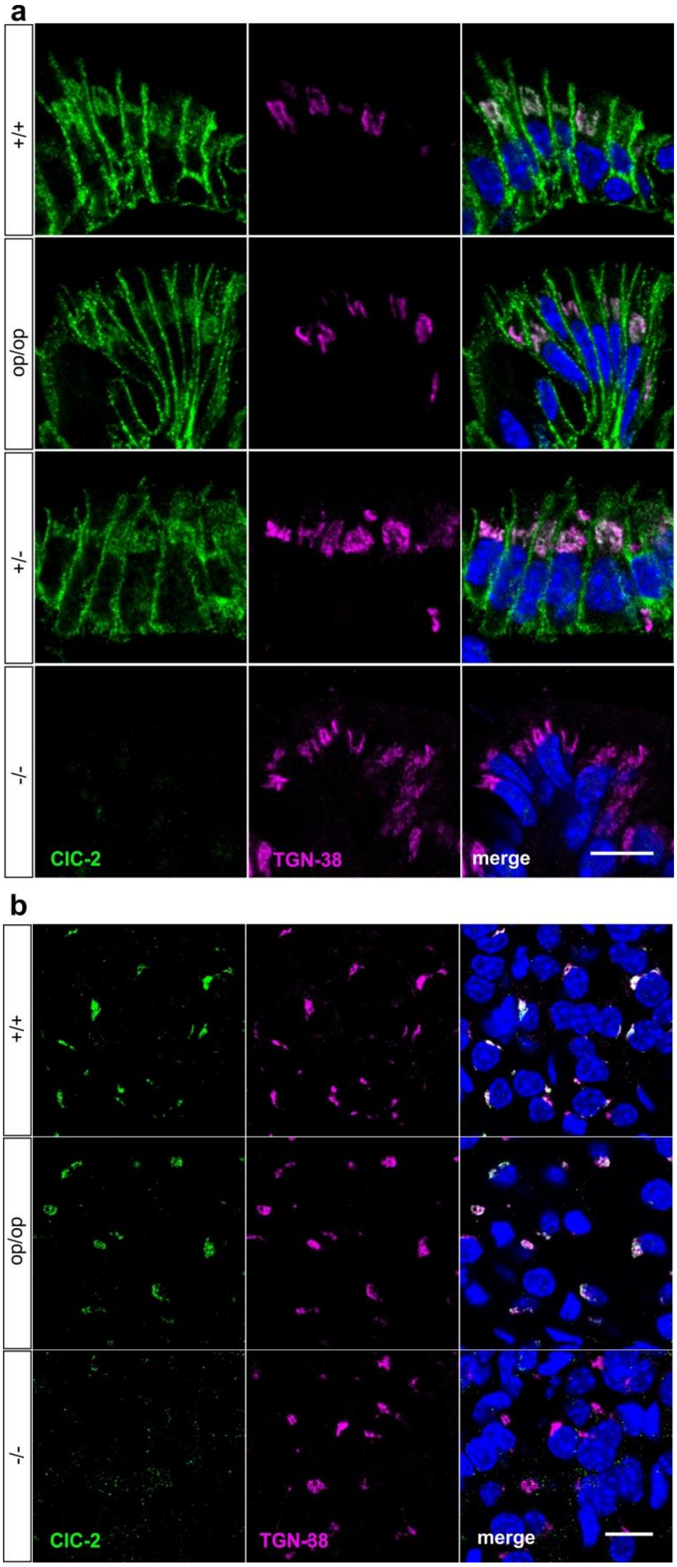

Supplementary Figure 3. Subcellular localization of mutant 'open' (op) and WT (+/+) ClC-2 protein. **a** Immunofluorescent staining of ClC-2 and the trans-Golgi network marker TGN-38 in the distal colon of 13 week-old female *Clcn2*$^{+/+}$ (+/+), *Clcn2*$^{op/op}$ (op/op), *Clcn2*$^{+/-}$ (+/-) and *Clcn2*$^{-/-}$ (-/-) mice and **b** in the zona glomerulosa of the adrenal gland of 12 week-old male *Clcn2*$^{+/+}$, *Clcn2*$^{op/op}$ and *Clcn2*$^{-/-}$ mice. **a**, **b** As staining patterns were comparable in male and female, images of only one sex are shown. Staining of *Clcn2*$^{+/-}$ mice **(a)** was performed to examine mono-allelic expression of *Clcn2*. Staining of *Clcn2*$^{-/-}$ tissue controls for antibody specificity. Scale bar, 10 µm.

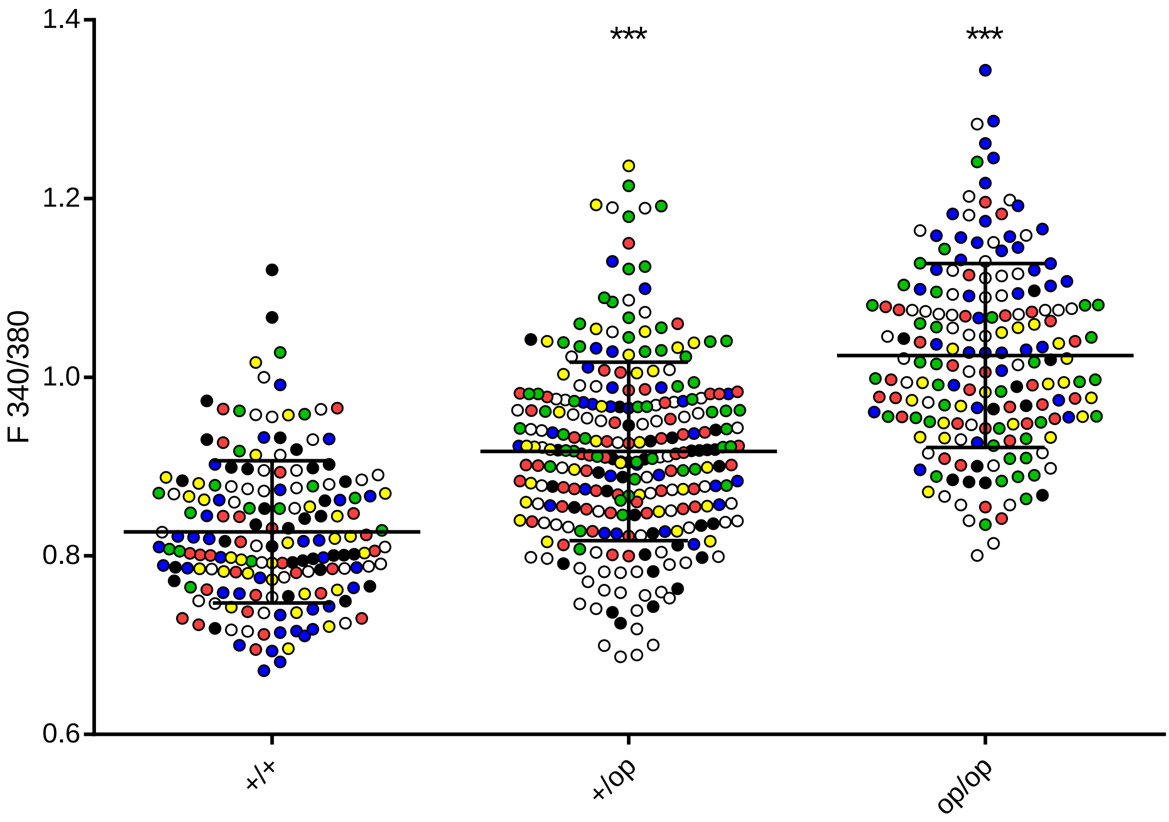

Supplementary Figure 4. Increased intracellular calcium in *Clcn2*^{op} zona glomerulosa cells. Data from all individual cells measured in the experiments of Fig. 4. Quantification of zona glomerulosa cells Ca^{2+} fluorescence from *Clcn2*$^{+/+}$ (+/+, n = 173 cells), *Clcn2*$^{+/op}$ (+/op, n = 267 cells) and *Clcn2*$^{op/op}$ (op/op, n = 182 cells) adrenal gland slices (6 mice analyzed for each genotype). Within each group, each color represents one animal. Error bars, mean ± SD. *** $p < 0.001$, +/op and op/op each compared to +/+, linear mixed effects model and pairwise comparisons (see Methods). Values are expressed in arbitrary units.

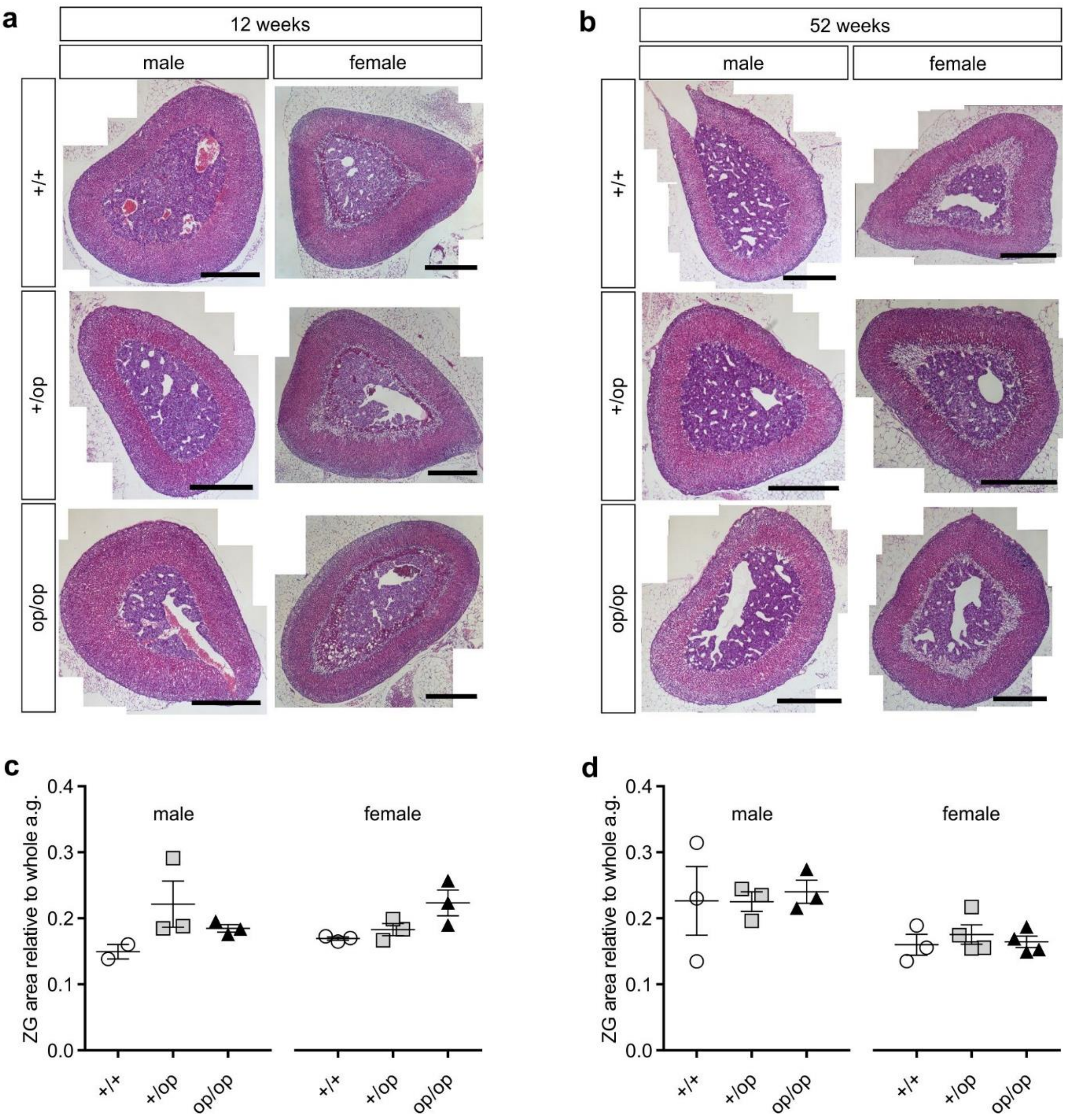

Supplementary Figure 5. Adrenal morphology is unchanged in *Clcn2*^{op} mice. **a, b** Hematoxylin eosin (HE) staining of paraffin-embedded adrenal gland slices from male and female *Clcn2*$^{+/+}$, *Clcn2*$^{+/op}$, *Clcn2*$^{op/op}$ mice designated at +/+, +/op, op/op at the age of 12 (**a**) and 52 weeks (**b**). Images are representative of 2-4 mice (see **c** and **d** for exact *n* number). Scale bars: 500 µm. **c, d** Quantification of the area of the zona glomerulosa (ZG) for (**a**, **b**) relative to the whole adrenal gland (a.g.) area using ImageJ. Each point represents one animal (mean of 3-4 slices per animal), reported as mean ± SEM.

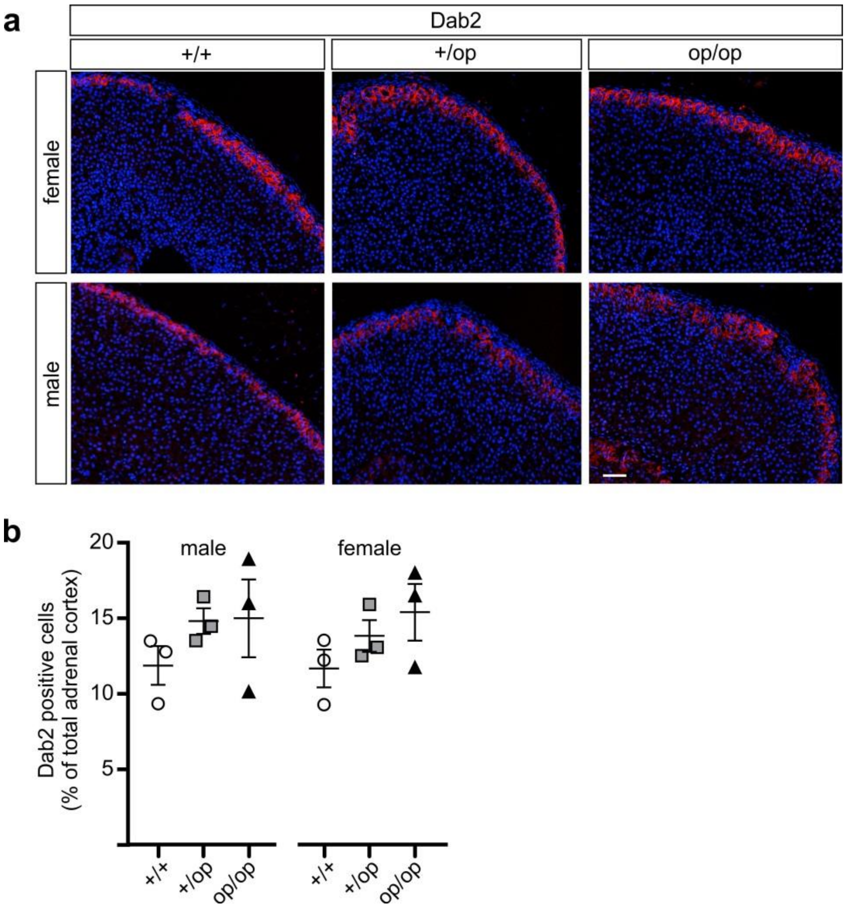

Supplementary Figure 6. Analysis of zona glomerulosa using Dab2 immunostaining. **a** Staining of paraffin-embedded adrenal gland slices from male and female *Clcn2*$^{+/+}$ (+/+, circles), *Clcn2*$^{+/op}$ (+/op, squares) and *Clcn2*$^{op/op}$ (op/op, triangles) mice at age 12 weeks for Dab2, a marker protein of ZG cells[4]. **b** Quantification of Dab2 positive cells as a percentage of total adrenal cortex of (**a**) experiments using an automated molecular imaging platform (Vectra, Perkin Elmer). n = 3 animals per group. Reported as mean ± SEM. No significant differences were found by performing statistical analysis using one-way-ANOVA, followed by Bonferroni multiple comparison test. Scale bar, 100 µm.

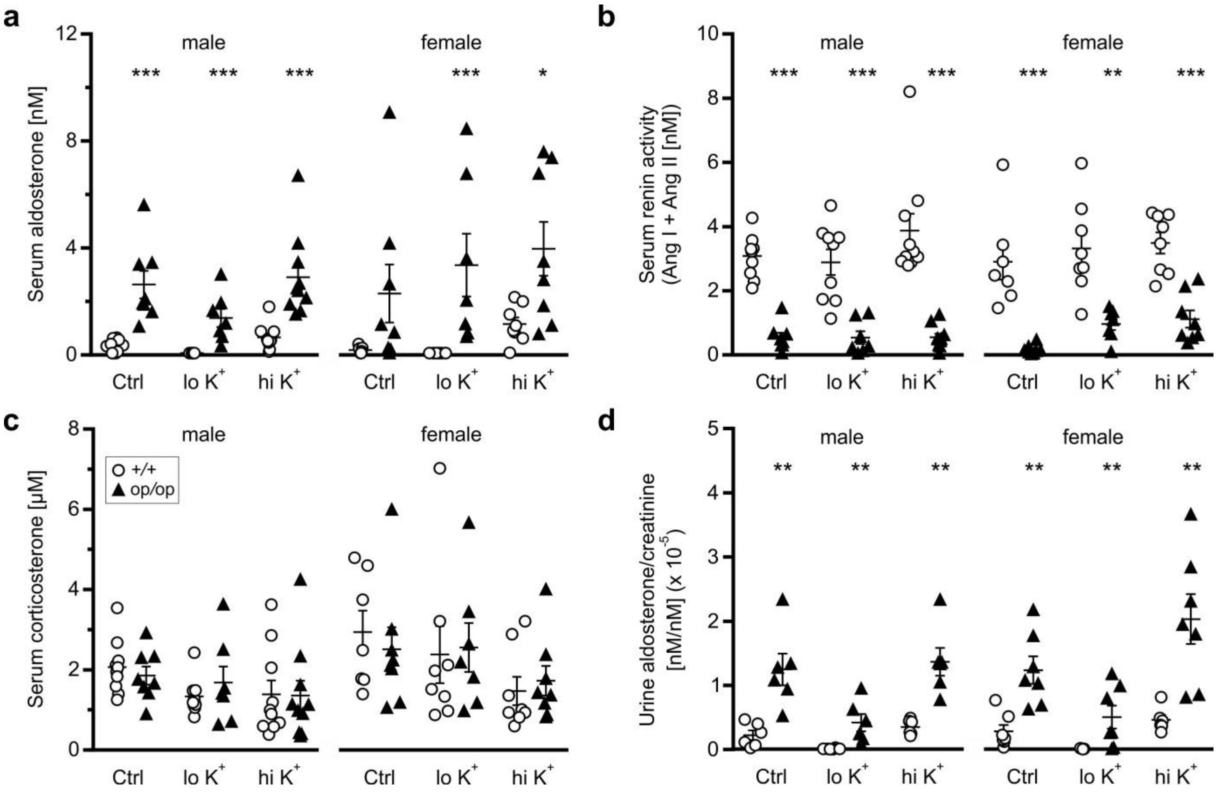

Supplementary Figure 7. Hormone levels in serum and urine of *Clcn2*$^{op/op}$ mice. **a-c** Serum aldosterone (**a**) and corticosterone (**c**) levels and renin activity (**b**) of *Clcn2*$^{op/op}$ (op/op, triangles) compared to *Clcn2*$^{+/+}$ (+/+, circles) mice (aged 20-31 weeks) fed with control (Ctrl), low potassium (lo K^+) or high potassium (hi K^+) diet and sub-grouped between males (left) and females (right)(each point represents one animal). Aldosterone levels below detection were set at the measureable threshold value of 70 pM (number of samples below threshold per samples measured: males, Ctrl: *Clcn2*$^{+/+}$ 3/10, *Clcn2*$^{op/op}$ 0/8; males, lo K^+: *Clcn2*$^{+/+}$ 9/9, *Clcn2*$^{op/op}$ 0/7; males, hi K^+: *Clcn2*$^{+/+}$ 0/10, *Clcn2*$^{op/op}$ 0/10; females, Ctrl: *Clcn2*$^{+/+}$ 2/7, *Clcn2*$^{op/op}$ 1/8; females, lo K^+: *Clcn2*$^{+/+}$ 8/8, *Clcn2*$^{op/op}$ 0/7; females, hi K^+: *Clcn2*$^{+/+}$ 0/8, *Clcn2*$^{op/op}$ 0/8). Renin activity was calculated as the sum of Ang I (Angiotensin I) and Ang II (Angiotensin II) (see methods). **d** 24h-urine aldosterone levels normalized to creatinine of *Clcn2*$^{op/op}$ (op/op, triangles) compared to *Clcn2*$^{+/+}$ (+/+, circles) mice (aged 20-31 weeks) fed with control (Ctrl), low potassium (lo K^+) or high potassium (hi K^+) diet and sub-grouped between males (left) and females (right)(each point represents one animal). Urine aldosterone levels below detection were set at the measureable threshold value of 0.2 nM (number of samples below threshold per samples measured: males, Ctrl: *Clcn2*$^{+/+}$ 0/6, *Clcn2*$^{op/op}$ 0/6; males, lo K^+: *Clcn2*$^{+/+}$ 6/6, *Clcn2*$^{op/op}$ 0/6; males, hi K^+: *Clcn2*$^{+/+}$ 0/6, *Clcn2*$^{op/op}$ 0/6; females, Ctrl: *Clcn2*$^{+/+}$ 0/7, *Clcn2*$^{op/op}$ 0/7; females, lo K^+: *Clcn2*$^{+/+}$ 7/7, *Clcn2*$^{op/op}$ 2/7; females, hi K^+: *Clcn2*$^{+/+}$ 0/6, *Clcn2*$^{op/op}$ 0/7). **a-d** Error bars, mean ± SEM. Statistical significance with reference to +/+ of each sex and diet is shown above the groups: * $p < 0.05$, ** $p < 0.01$, *** $p < 0.001$ (Mann-Whitney *U* test).

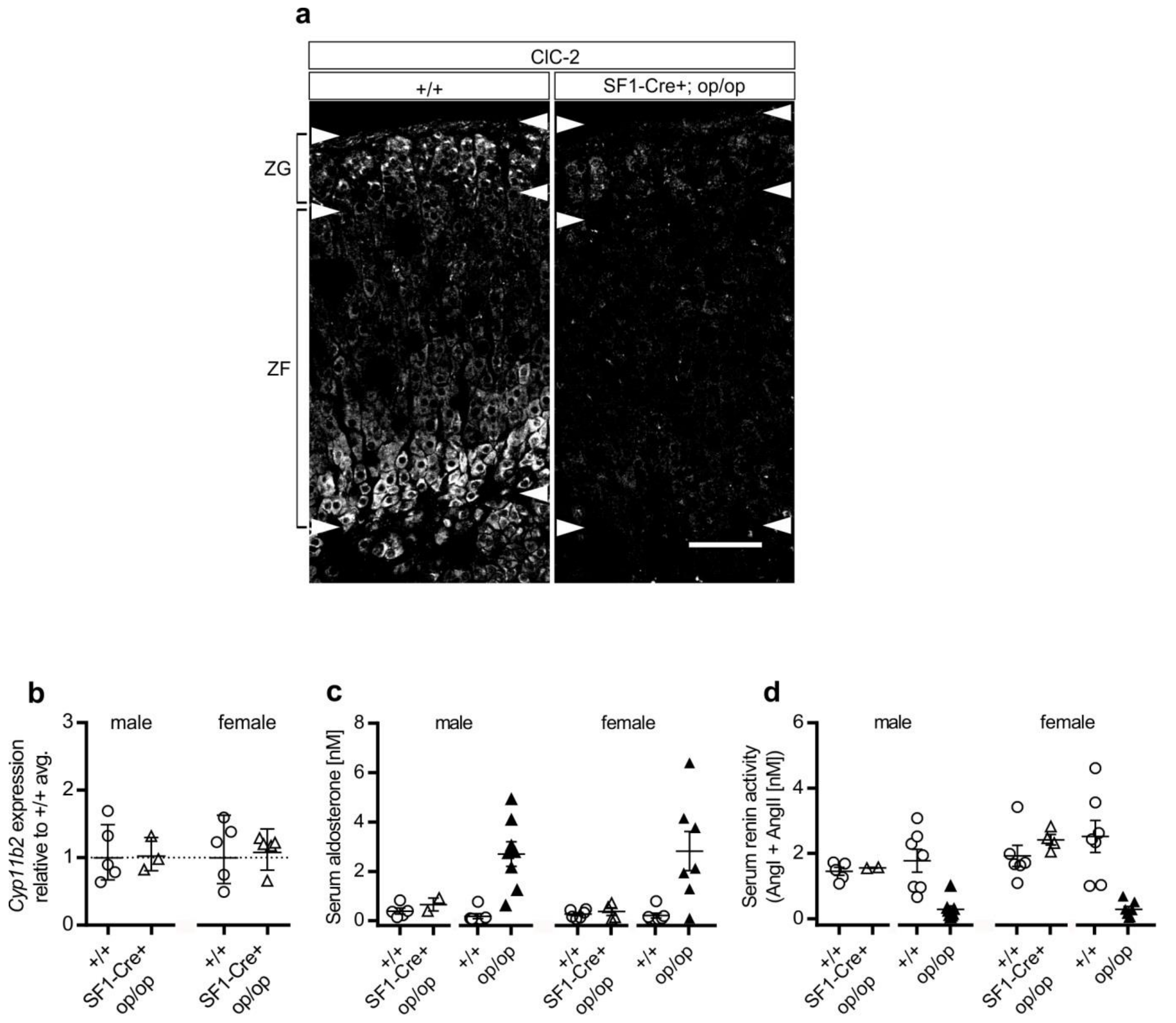

Supplementary Figure 8. *Adrenal cortex-specific deletion of Clcn2^{op} prevents primary aldosteronism.* **a** Immunofluorescent staining of ClC-2 in adrenal cortex of 25 week-old female *Clcn2*$^{+/+}$ (+/+) and SF1-Cre+; *Clcn2*$^{op/op}$ (SF1-Cre+; op/op) mice. White arrowheads indicate approximate boundaries of zona glomerulosa (ZG) and zona fasciculata (ZF) Scale bar, 50 µm. **b** Quantitative RT-PCR analysis of *Cyp11b2* mRNA expression in adrenal glands from 13-26 week-old male and female *Clcn2*$^{+/+}$ (+/+, circles) and SF1-Cre+; *Clcn2*$^{op/op}$ (SF1-Cre+; op/op, triangles), $n \geq 3$ (each point represents one animal). Relative expression levels compared to *Clcn2*$^{+/+}$ average. Error bars, geometric mean ± geometric SD. No significant difference was found using Mann-Whitney U test. **c, d** Serum levels of aldosterone (**c**) and renin activity (**d**) of *Clcn2*$^{+/+}$ (+/+, circles) and SF1-Cre+; *Clcn2*$^{op/op}$ (SF1-Cre+; op/op, white triangles) (age: 13-26 weeks) compared to *Clcn2*$^{+/+}$ (+/+, open circles) and *Clcn2*$^{op/op}$ (op/op, black triangles; values for +/+ and op/op are the same as in Fig. 6 and shown here for comparison; each point represents one animal). Aldosterone levels below detection limit were set at the measureable threshold value of 70 pM (number of samples below threshold per samples measured: males: *Clcn2*$^{+/+}$ n=0/5, SF1-Cre+; *Clcn2*$^{op/op}$ n=0/2; females: *Clcn2*$^{+/+}$ n=1/6, SF1-Cre+; *Clcn2*$^{op/op}$ n=1/4). Renin activity was calculated as the sum of Ang I and Ang II. Error bars, mean ± SEM.

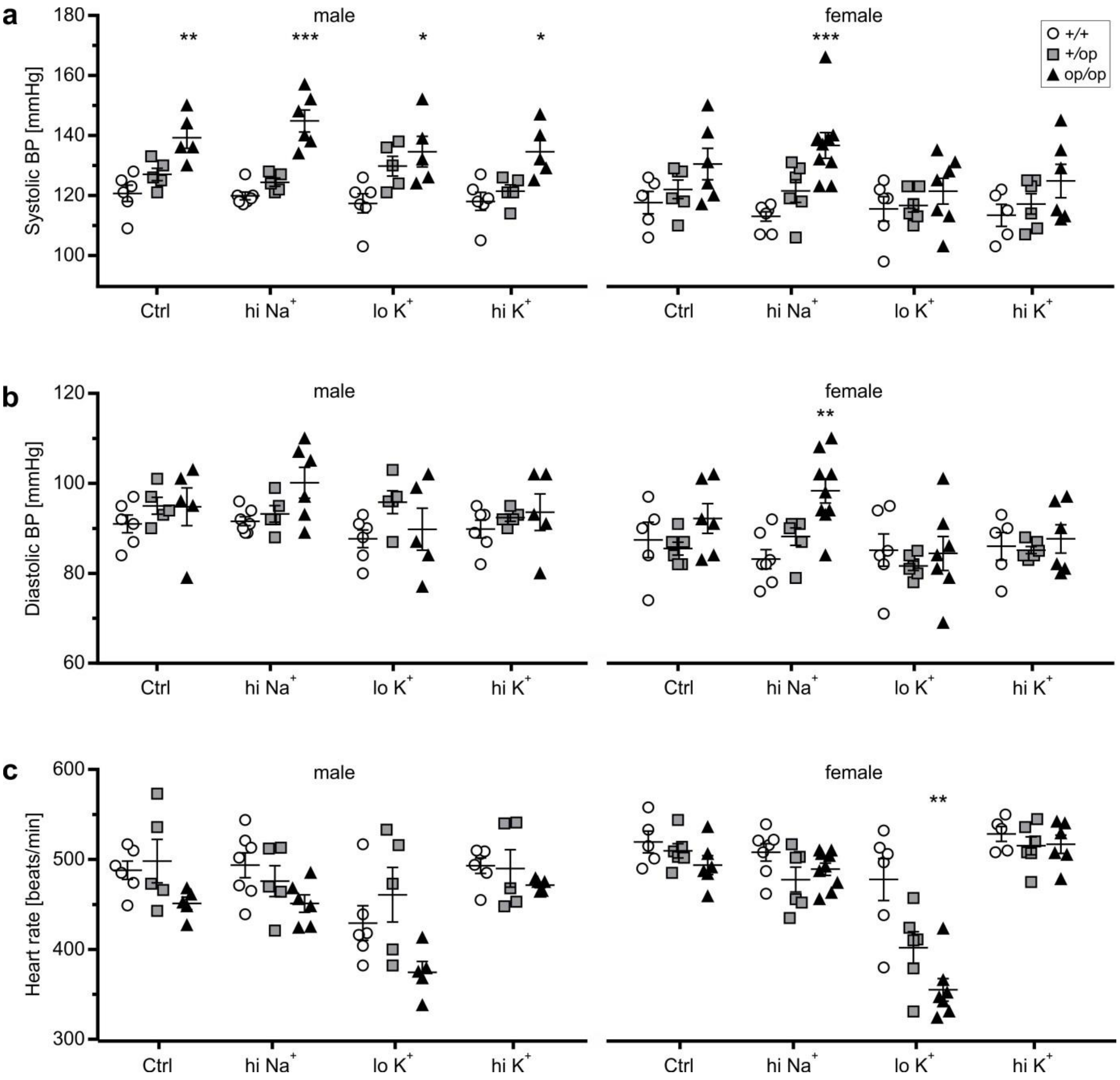

Supplementary Figure 9. Changes in blood pressure and heart rate in male and female *Clcn2*$^{op/op}$ mice. **a-c** Systolic (**a**) and diastolic (**b**) blood pressure and heart rate (**c**) measured by telemetry in *Clcn2*$^{+/op}$ (+/op, grey squares) and *Clcn2*$^{op/op}$ mice (op/op, black triangles) mice compared to *Clcn2*$^{+/+}$ (+/+, white circles) fed with control (Ctrl), high sodium (hi Na^+), low potassium (lo K^+) or high potassium (hi K^+) diet (n = 5-9 each point represents one animal). Males and females were analyzed separately. Single values represent mean values of one mouse measured during seven consecutive days. Error bars, mean ± SEM. Statistical significance with reference to +/+ of each sex and diet is shown above the groups: * $p < 0.05$, ** $p < 0.01$, *** $p < 0.001$ (Kruskal-Wallis test, Dunn's multiple comparison test).

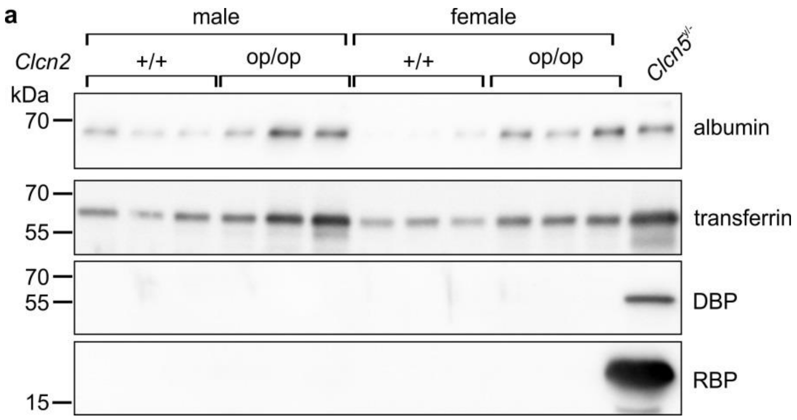

Supplementary Figure 10. Mild albuminuria in *Clcn2$^{op/op}$* mice. 24h-urine samples from 22-29 week-old male and female *Clcn2$^{+/+}$* (+/+) and *Clcn2*$^{op/op}$ (op/op) mice were normalized to creatinine and analyzed by Western blot for albumin, transferrin, vitamin D binding protein (DBP) and retinol binding protein (RBP). Urine from mice lacking (X-chromosomal) ClC-5 (*Clcn5*$^{y/-}$) was used as control as these mice show low molecular weight proteinuria (as indicated by the presence of DBP and RBP in urine) as well as increased albumin and transferrin levels[5]. *Clcn2*$^{op/op}$ mice show increased albumin as well as transferrin levels, but not low MW proteinuria. Western blot is representative of *n* = 6 animals analyzed per genotype and sex.

Supplementary Table 1. Blood electrolyte concentrations and pH of *Clcn2*$^{+/+}$ and *Clcn2*$^{op/op}$ mice with control, low potassium (lo K^+) or high potassium (hi K^+) diet.

Diet	sex	genotype	*n*	Na^+	K^+	Cl^-	pH	HCO_3^-
ctrl	m	*Clcn2*$^{+/+}$	11	149.2±0.8	3.7±0.2 [0/11]	113.3±1.6	7.26±0.04	23.0±2.5
ctrl	m	*Clcn2*$^{op/op}$	9	149.4±0.9	3.1±0.2 [0/9]***	111.3±3.0	7.28±0.11	23.2±4.4
lo K^+	m	*Clcn2*$^{+/+}$	9	152.2±1.1	<2.6±0.4 [1/9]	114.1±1.1	7.29±0.04	25.1±1.7
lo K^+	m	*Clcn2*$^{op/op}$	9	151.2±1.5	<2.1±0.2 [6/9]**	101.1±5.8****	7.44±0.06***	37.5±8.4**
hi K^+	m	*Clcn2*$^{+/+}$	4-7	149.7±0.8	3.3±0.6 [0/7]	112.7±1.6	7.26±0.07	22.3±3.9
hi K^+	m	*Clcn2*$^{op/op}$	9-10	151.1±1.7*	3.2±1.0 [0/9]	108.5±1.8***	7.31±0.06	27.3±4.2
ctrl	f	*Clcn2*$^{+/+}$	9-10	148.8±1.1	3.4±0.4 [0/9]	114.6±2.5	7.24±0.07	20.5±2.3
ctrl	f	*Clcn2*$^{op/op}$	10	150.1±1.3*	2.7±0.2 [0/10]***	111.9±2.9	7.29±0.05	23.1±2.6*
lo K^+	f	*Clcn2*$^{+/+}$	10	153.0±1.9	<2.4±0.3 [2/10]	114.4±2.5	7.28±0.03	22.8±2.7
lo K^+	f	*Clcn2*$^{op/op}$	10	151.9±1.8	<2.0 [10/10]***	104.9±3.0****	7.38±0.07***	31.0±4.6***
hi K^+	f	*Clcn2*$^{+/+}$	9	150.7±1.3	3.8±0.3 [0/9]	113.0±3.9	7.26±0.05	22.7±3.2
hi K^+	f	*Clcn2*$^{op/op}$	9	152.1±2.0	3.0±0.4 [0/9]***	111.8±3.2	7.32±0.05*	25.6±3.4

m, male. f, female. Ion concentrations in mM. *n*, number of mice. Mean values ± SD; For K^+ column in parenthesis: number of values below the detection threshold (2 mM) per samples measured, these values were set as "2"; significant difference with reference to *Clcn2*$^{+/+}$ of the same sex and fed with the same diet: * $p < 0.05$ ** $p < 0.01$ **** $p < 0.0001$ (Mann-Whitney *U* test).

Supplementary References:

1. Blanz, J. *et al.* Leukoencephalopathy upon disruption of the chloride channel ClC-2. *J Neurosci* **27**, 6581-6589 (2007).

2. Hoegg-Beiler, M. B. *et al.* Disrupting MLC1 and GlialCAM and ClC-2 interactions in leukodystrophy entails glial chloride channel dysfunction. *Nature Communications* **5**, 3475 (2014).

3. Catalán, M. *et al.* ClC-2 in guinea pig colon: mRNA, immunolabeling, and functional evidence for surface epithelium localization. *Am J Physiol Gastrointest Liver Physiol* **283**, G1004-G1013 (2002).

4. Romero, D.G. *et al.* Disabled-2 is expressed in adrenal zona glomerulosa and is involved in aldosterone secretion. *Endocrinology* **148,** 2644–2652 (2007).

5. Piwon, N. *et al.* ClC-5 Cl^--channel disruption impairs endocytosis in a mouse model for Dent's disease. *Nature* **408**, 369-373 (2000).
