# Supplementary material for: Pathogenesis of hypertension in a mouse model for human CLCN2 related hyperaldosteronism
Source: Nat Commun. 2019 Oct 15;10:4678. doi: 10.1038/s41467-019-12113-9 (PMC6794291; doi:10.1038/s41467-019-12113-9)
Supplement: Supplementary file 3 — Description of Additional Supplementary Files [file 41467_2019_12113_MOESM3_ESM.docx]

**Description of Additional Supplementary Files**

**File Name: Supplementary Movie 1**

**Description:** Calcium measurements in Clcn2+/+ (+/+) zona glomerulosa cells. Representative time lapse imaging video (sped up six times, real time: two minutes) of Fura2 fluorescence in Clcn2+/+ adrenal slices under basal conditions. Imaging was performed and analyzed as described in Fig. 4.

**File Name: Supplementary Movie 2**

**Description:** Calcium oscillations in Clcn2op/op (op/op) zona glomerulosa cells. Representative time lapse imaging video (sped up six times, real time: two minutes) of Fura2 fluorescence in Clcn2op/op adrenal slices under basal conditions. Imaging was performed and analyzed as described in Fig. 4
