# Supplementary material for: Pathogenesis of hypertension in a mouse model for human CLCN2 related hyperaldosteronism
Source: Nat Commun. 2019 Oct 15;10:4678. doi: 10.1038/s41467-019-12113-9 (PMC6794291; doi:10.1038/s41467-019-12113-9)
Supplement: Supplementary file 6 — Reporting Summary [file 41467_2019_12113_MOESM6_ESM.pdf]

nature research

Corresponding author(s): Thomas J Jentsch

Last updated by author(s): 2019/6/24

Reporting Summary

Nature Research wishes to improve the reproducibility of the work that we publish. This form provides structure for consistency and transparency in reporting. For further information on Nature Research policies, see Authors & Referees and the Editorial Policy Checklist.

Statistics

For all statistical analyses, confirm that the following items are present in the figure legend, table legend, main text, or Methods section.

n/a	Confirmed	
☐	☒	The exact sample size (n) for each experimental group/condition, given as a discrete number and unit of measurement
☐	☒	A statement on whether measurements were taken from distinct samples or whether the same sample was measured repeatedly
☐	☒	The statistical test(s) used AND whether they are one- or two-sided<br>*Only common tests should be described solely by name; describe more complex techniques in the Methods section.*
☐	☒	A description of all covariates tested
☐	☒	A description of any assumptions or corrections, such as tests of normality and adjustment for multiple comparisons
☐	☒	A full description of the statistical parameters including central tendency (e.g. means) or other basic estimates (e.g. regression coefficient) AND variation (e.g. standard deviation) or associated estimates of uncertainty (e.g. confidence intervals)
☐	☒	For null hypothesis testing, the test statistic (e.g. F, t, r) with confidence intervals, effect sizes, degrees of freedom and P value noted<br>*Give P values as exact values whenever suitable.*
☒	☐	For Bayesian analysis, information on the choice of priors and Markov chain Monte Carlo settings
☒	☐	For hierarchical and complex designs, identification of the appropriate level for tests and full reporting of outcomes
☒	☐	Estimates of effect sizes (e.g. Cohen's d, Pearson's r), indicating how they were calculated

Our web collection on statistics for biologists contains articles on many of the points above.

Software and code

Policy information about availability of computer code

Data collection	Clampex 10.6 (Molecular Devices), MetaFluor 7.10 (Molecular Devices), ZEN 2.3 (Zeiss), Vectra® automated imaging system (Perkin Elmer), Dataquest A.R.T. (DSI), StepOne Software 2.3 (Life Technologies)
Data analysis	ClampFit 10.6 (Molecular Devices), MetaFluor 7.10 (Molecular Devices), ImageJ 1.52a (NIH), Prism 7 (Graphpad), Sigma plot 10 (Systat), ZEN 2.3 (Zeiss), InForm (Perkin Elmer), R 3.4.3 (R Core Team), StepOne Software 2.3 (Life Technologies)

For manuscripts utilizing custom algorithms or software that are central to the research but not yet described in published literature, software must be made available to editors/reviewers. We strongly encourage code deposition in a community repository (e.g. GitHub). See the Nature Research guidelines for submitting code & software for further information.

Data

Policy information about availability of data

All manuscripts must include a data availability statement. This statement should provide the following information, where applicable:

- Accession codes, unique identifiers, or web links for publicly available datasets
- A list of figures that have associated raw data
- A description of any restrictions on data availability

The data that support the findings of this study are available from the corresponding author upon reasonable request.

Field-specific reporting

Please select the one below that is the best fit for your research. If you are not sure, read the appropriate sections before making your selection.

☒ Life sciences ☐ Behavioural & social sciences ☐ Ecological, evolutionary & environmental sciences

Life sciences study design

All studies must disclose on these points even when the disclosure is negative.

Sample size	Sample sizes chosen for electrophysiologcal studies (Fig. 1,3) were based on a previous publication characterizing a CLCN2 disease mutation (Fernandes-Rosa et al, Nat Genet 2018) and N-terminal mutations (Gründer et al, Nature 1992). Samples sizes were deemed sufficient based on the clearly visible effects of the mutations on the mean current, mean membrane potential and the overall distribution of data points within and between each group. The effects seen on membrane potential in adrenal cells and the number of cells measured are similar to what has been reported in previous studies (Davies et al, PNAS 2008; Penton et al, Endocrinology 2012). For calcium imaging in zona glomerulosa cells (Fig. 4), sample sizes were chosen based on the previous study of Penton et al, Endocrinology 2012 and was sufficient based on the clear differences observed between genotypes. Based on the reviewer's comments, we have now increased the number of animals to 6 per genotype.<br>Sample size of mice used for blood pressure and serum aldosterone measurements was determined using G*power a priori power analysis, which was based on empirical values provided by specialists performing telemetry at our institute and for hormone measurements by Attoquant GmbH. Tissue of the same mice used for aldo/BP measurements were conveniently used for qRT-PCR, Western blot analysis, histology and immunofluorescence stainings.
Data exclusions	For TEVC experiments, controls experiments were performed with non-injected oocytes and if leak currents were detected the entire batch of oocytes was discarded (pre-established criteria). For perforated patch clamp measurements, cells were excluded when seals were unstable or lost (abrupt decrease in membrane current) during perforation (pre-established criteria). For calcium imaging, slices were excluded which underwent movement during time lapse imaging or were too blurry (pre-established criteria). For morphological analysis of adrenal glands (Sup Fig. 4), sections were discarded when tissue was torn/badly damaged during preparation.<br>For qRT-PCR experiments, all samples were run as triplicates and mean Ct values were used for calculations. If the Ct values of 1 out of the 3 wells deviated more than 0.5 Ct from the other 2, it was excluded from analyses and calculations were performed with the remaining 2 wells.
Replication	For TEVC and patch clamp analysis of mutations, measurements were performed in several batches and transfections, respectively. For patch clamp measurements of zona glomerulosa cells, 1-2 cells were measured per animal, and animals were measured on different days with new bath solutions prepared each time.<br>qRT-PCR data were collected in 3 independent sets of experiments, all samples were run as triplicates.<br>Groups of 7-11 mice were used for serum aldosterone and blood electrolyte measurements, groups of 5-9 mice were used for blood pressure measurement. Western blots were repeated with organs of min. three mice per genotype.
Randomization	For randomization of patch clamp analysis and calcium imaging in adrenal slices, analysis of animals of different genotypes was interleaved.<br>For all other experiments, control (WT) and experimental (knock-in mouse model) were always performed together.
Blinding	Blinding was not performed and was unnecessary since most measurements were quantitative.

Reporting for specific materials, systems and methods

We require information from authors about some types of materials, experimental systems and methods used in many studies. Here, indicate whether each material, system or method listed is relevant to your study. If you are not sure if a list item applies to your research, read the appropriate section before selecting a response.

Materials & experimental systems

n/a	Involved in the study	
☐	☒	Antibodies
☐	☒	Eukaryotic cell lines
☒	☐	Palaeontology
☐	☒	Animals and other organisms
☒	☐	Human research participants
☒	☐	Clinical data

Methods

n/a	Involved in the study	
☒	☐	ChIP-seq
☒	☐	Flow cytometry
☒	☐	MRI-based neuroimaging

Antibodies

Antibodies used	ClC-2 (rabbit anti-mouse raised against C-terminal peptide of mouse ClC-2 ((C)WGPRSRHGLPREGTPSDSDDKSQ); NKCC1 (rabbit anti-mouse raised against peptide CDEEEDGKTPTQPLL); TGN-38 (AHP499, BioRad, 1:200 for IHC); Dab2 (1:100 for IHC, sc-13982, Santa Cruz Biotechnology); actin (clone AC-74, Sigma, A2228, 1:1,000 for WB); albumin (Biotrend, 1:5000 for WB); transferrin (Immunovision, 1:250 for WB); vitamin D binding protein ([DBP] Biorad, 9580-2710, 1:300 for WB); retinol binding protein [RBP] (Rabbit anti retinol binding protein (RBP) was a generous gift from Bill Blaner (Columbia University, NY, USA) and has been used in our previous studies (Piwon et al., Nature 2000; Novarino et al. Science 2010); Rabbit anti Cyp11b2 (1:25 for IHC) was a generous gift from CE Gomez-Sanchez
Validation	The specificity of the ClC-2 antibody in this study (Fig 2, Sup Fig 3, Western blot, and immunostaining) was verified using the ClC-2 KO tissue, both in a previous study (Hoegg-Beiler et al., Nature Commun. 2014) and in the present work. The specificity of the NKCC1 antibody has been previously tested against various tissues from KO animals, and in the present study in IHC of

adrenal glands from Nkcc1-/- mice. The Cyp11b2 and Dab2 immunostaining pattern in zona glomeruolsa cells observed in the present work is consistent with the literature. The TGN38 antibody has been shown to label the trans Golgi network of murine cells (see manufacturer's website). The DBP antibody was raised against human DBP (see manufacturer's website) and detects a band of correct size in urine only from mice showing low MW proteinuria. Generation and specificity testing of RBP antibody was described (Kato et al., PNAS, 1985;82(8):2488-92).

Eukaryotic cell lines

Policy information about cell lines

Cell line source(s)	HEK293 (ATCC)
Authentication	No authentication performed.
Mycoplasma contamination	N/A
Commonly misidentified lines (See ICLAC register)	*Name any commonly misidentified cell lines used in the study and provide a rationale for their use.*

Animals and other organisms

Policy information about studies involving animals; ARRIVE guidelines recommended for reporting animal research

Laboratory animals	Mice (mixed background, between 3 and 52 weeks of age, both male and females); Xenopus leaevis frogs (pigmented, albino) used for harvesting oocytes were 6-18 months of age
Wild animals	Study did not involve wild animals.
Field-collected samples	Study did not involve field-collected animals.
Ethics oversight	LAGeSo Berlin, Germany

Note that full information on the approval of the study protocol must also be provided in the manuscript.
